# A Retrospective Cohort Study of Myosteatosis and Quality of Life in Head and Neck Cancer Patients

**DOI:** 10.3390/cancers13174283

**Published:** 2021-08-25

**Authors:** Amy L. Shaver, Katia Noyes, Heather M. Ochs-Balcom, Gregory Wilding, Andrew D. Ray, Sung Jun Ma, Mark Farrugia, Anurag K. Singh, Mary E. Platek

**Affiliations:** 1 Department of Epidemiology and Environmental Health, School of Public Health and Health Professions, University at Buffalo, Buffalo, NY 14214, USA; enoyes@buffalo.edu (K.N.); hmochs2@buffalo.edu (H.M.O.-B.); 2 Department of Radiation Medicine, Roswell Park Comprehensive Cancer Center, Buffalo, NY 14203, USA; SungJun.Ma@RoswellPark.org (S.J.M.); Mark.Farrugia@RoswellPark.org (M.F.); Anurag.Singh@RoswellPark.org (A.K.S.); Mary.Platek@RoswellPark.org (M.E.P.); 3 Department of Medical Oncology, Sidney Kimmel Cancer Center at Jefferson, Sidney Kimmel Medical College, Philadelphia, PA 19107, USA; 4 Department of Biostatistics, School of Public Health and Health Professions, University at Buffalo, Buffalo, NY 14214, USA; gwilding@buffalo.edu; 5 Department of Cancer Prevention and Control, Roswell Park Comprehensive Cancer Center, Buffalo, NY 14203, USA; andrew.ray@roswellpark.org; 6 Department of Rehabilitation Sciences, Roswell Park Comprehensive Cancer Center, Buffalo, NY 14203, USA; 7 Department of Dietetics, D’Youville College, Buffalo, NY 14203, USA

**Keywords:** Myosteatosis, head and neck cancer, quality of life, definitive treatment, body composition

## Abstract

**Simple Summary:**

Quality of life (QOL) is an important patient reported outcome that effects both care and life outside of treatment. There is a shortage of nontaxing ways to determine which patients may need enhanced care over the course of their therapy and beyond to help avoid long-term declines in QOL. Therefore, we investigated whether myosteatosis as determined through existing diagnostic imaging could be used to predict QOL trajectories. In this study, patients with pretreatment myosteatosis were more likely to have lower physical and global QOL scores than patients with normal muscle density. In conclusion, myosteatosis may be a way of determining patients in need of extra assistance over the course of treatment and afterwards.

**Abstract:**

Head and neck cancer (HNC) treatment-related morbidity can be detrimental to quality of life (QOL). Myosteatosis is associated with poor QOL in multiple cancers. If predictive of poor QOL trajectories, myosteatosis would be a tool for clinicians to determine which patients may require additional support during treatment. The purpose of this study was to determine if pretreatment myosteatosis is associated with a poor QOL trajectory following treatment completion. Methods: In a retrospective cohort design, myosteatosis was determined from pretreatment CT scans. Both physical and global QOL score was assessed through patient interview on follow-up appointment. Demographic, cancer-specific, and social covariates were collected, reported, and considered as potential confounders. Results: The population of 163 patients was mostly male (82.2%) and white (91.4%) with oropharyngeal cancer (55.8%). Males with myosteatosis had a physical QOL score 46.84 points lower at one-year following treatment completion (*p* = 0.01) than those with normal muscle density (*p* = 0.01). Males with myosteatosis averaged 57.57 points lower at one-year post-treatment (*p* = 0.01) in global QOL scores. Conclusions: Over one year following completion of treatment, patients with myosteatosis reported worse physical and global QOL scores than patients with normal muscle density.

## 1. Introduction

Quality of life (QOL) is a patient-reported outcome that is important to measure both during and after cancer care since QOL is an important measure of quality of care [1]. QOL is a multidimensional construct that refers to a patient’s perceived mental and physical health status [2]. QOL can be measured via validated questionnaire and refers to multiple aspects of a patient’s life including physical, emotional, social, and cognitive functions. The questionnaires also provide patients the opportunity to provide an overall, or global, QOL score. As survival times improve in patients with head and neck cancer (HNC) the trajectory of QOL becomes increasingly important [3]. QOL has been associated with survival outcomes in HNC patients [4].

Sarcopenia, a syndrome characterized by progressive and generalized loss of skeletal muscle mass and strength, is highly prevalent in the HNC population and is associated with poor overall survival [5,6,7]. A recent meta-analysis indicated the cumulative prevalence at 42.0% [8]. Myosteatosis is the fatty infiltration of muscle tissue. It can be visualized as low skeletal muscle density (SMD) on CT scan and is a contributing element to sarcopenia [9]. Findlay et al. indicated that sarcopenia and myosteatosis were associated with both financial toxicity in HNC patients as well as higher unplanned hospitalization costs [10]. Myosteatosis has been associated with QOL in other cancer types.

A study in non-small-cell lung cancer indicated that those with lower SMD (compared to normal SMD) had worse physical function QOL, particularly in relation to dyspnea [11]. A study among early-stage colorectal cancer patients indicated that both low skeletal muscle mass and low SMD was associated with increased fatigue and decreased QOL [12]. Myosteatosis has not been studied in association with QOL among HNC patients. However, associations have been found in other cancer types. The association between myosteatosis and QOL has not been studied in HNC patients. If predictive of QOL trajectories in HNC patients, SMD may serve as a useful tool to aid in determining which patients require additional support services (such as prehabilitation or post-treatment rehabilitation) over the course of and following their cancer treatment in order to improve patient outcomes and decrease the occurrence of adverse effects. In this way, SMD could serve as a marker of physical resilience.

The objective of this study was to evaluate the association of pretreatment myosteatosis with QOL trajectory in HNC survivors over 12 months. We hypothesized that myosteatosis was predictive of poor global and physical QOL score from pretreatment through one year after treatment completion.

## 2. Materials and Methods

### 2.1. Study Design and Population

Longitudinal analyses were utilized to examine the association between skeletal muscle density (SMD) and quality of life both at baseline and over 12 months following treatment completion in a sample of HNC patients. Data from HNC patients over 18 years of age treated at Roswell Park Comprehensive Cancer Center (RPCCC) from 2008 to 2017 was utilized for the study. We examined SMD as a marker of physical resilience and determined its association with global and physical function QOL scores. The RPCCC Institutional Review Board approved the study.

Individuals treated for squamous cell HNC using definitive radiation therapy (RT) and treated by one radiation oncologist at RPCCC were considered for inclusion in this study. Those without readable computerized tomography (CT) scans of the third lumbar (L3) vertebral body and missing QOL questionnaires both before and after treatment were excluded. At RPCCC those who receive definitive RT routinely receive CT as part of both clinical practice and institutional guideline. Those with triple therapy (e.g., CCRT and surgery) were also excluded.

### 2.2. Marker Measurement: Muscle and Adipose Tissue

Imaging software (SliceOmatic Software, version 5.0, TomoVision, Magog, QC, Canada) was used to quantify the cross-sectional area of muscle (a measure of skeletal muscle mass) and adipose tissue in centimeters squared at L3. One researcher, who was blinded to clinical data, performed CT interpretation using the imaging software to estimate SMD and other body composition measures. Subsequently, three independent researchers audited the original estimations by reanalyzing a random 20% sample of CT images. The results were all within a margin of ±5.0%. The imaging software allows for measurement of skeletal muscle, visceral adipose tissue, subcutaneous adipose tissue, and intermuscular adipose tissue through the use of tissue-specific Hounsfield units (HU) ranges to determine different tissue types, as has been previously described [13]. The L3 level is used in studies of body composition because the estimates of whole-body volume determined from that area have been previously validated [14]. A measure of skeletal muscle mass, skeletal muscle index (SMI), was created by adjusting muscle area for patient height (calculated by dividing the muscle area at L3 by patient height in meters squared). This adjustment is completed to enable comparisons between subjects. IMAT (intermuscular adipose tissue) produces less radiation attenuation than muscle without adipose tissue; therefore, the adipose tissue causes the muscle to appear less dense and to have a lower average HU reading. Skeletal muscle radiodensity (SMD), as measured by the mean radiation attenuation in HU, was used as the measure of muscle density. 

SliceOmatic was also used to quantify adipose tissue in centimeters squared at the L3 level using the same method as described above [13]. Total adipose tissue (TAT) area at L3 in cm^2^ was constructed through addition of visceral adipose tissue (VAT), subcutaneous adipose tissue (SAT) and IMAT and each was reported.

### 2.3. Outcome Measurement: Quality of Life

Patients at RPCCC completed the European Organization for the Research and Treatment of Cancer Quality of Life questionnaire in Cancer (EORTC-QLQ-C30) which consists of 30 questions [15]. The EORTC-QLQ-C30 contains five functional scales (physical, social, role, cognitive, and emotional) as well as measures of financial impact, overall QOL and eight symptom scales (fatigue, nausea/vomiting, pain, dyspnea, sleep disturbances, appetite loss, constipation, and diarrhea). The questionnaire has been validated in multiple cancer patient populations [16]. This study utilized the item for global QOL and the physical function scale. The responses to the EORTC-QLQ-C30 questions were scored according to the EORTC scoring manual which scales scores to a 100-point scale [17]. Patients completed the EORTC-QLQ-C30 at each clinic visit after the nurses’ assessment had been completed and prior to the patient seeing the physician. Clinic visits occurred prior to treatment, at treatment completion, and at three, six, nine, and twelve months following treatment completion. Questionnaires were administered digitally on an iPad. Patients generally completed the questionnaire on their own, but were offered staff assistance if necessary. Results from the survey were uploaded to a Research Electronic Data Capture (REDCap) database in real time and were available to treating clinicians.

### 2.4. Covariate Parameterization and Possible Confounders

#### 2.4.1. Demographics

Demographic, diagnosis, treatment, and social history data were collected via retrospective review of the electronic medical record and from patient self-report. Demographic data included age, sex, and race. Age and sex were retrieved from the medical record while race was self-reported as one of the following categories: White, Black, Native American or Alaskan Native (NAAN), and Asian or Pacific Islander (API). For the analysis, race was parameterized as White and Black, Indigenous, and Person of Color (BIPOC) in an effort to collapse smaller categories. 

#### 2.4.2. Diagnosis and Treatment

Diagnosis and treatment data were retrieved through medical record review. All patients were diagnosed with a primary occurrence of squamous cell head and neck cancer. Primary tumor sites collected included: oral cavity, nasopharynx, oropharynx, hypopharynx, laryngeal, salivary, other, and multiple. Tumor stage was collected and reported according to the American Joint Committee on Cancer (AJCC) staging criteria, seventh edition and parameterized as 0, I, II, III, and IV. For regression analysis, site was categorized as oropharyngeal, laryngeal, and other, and stage was categorized as stage I and II, stage III, and stage IV. Treatment included definitive radiation therapy (RT) or definitive concomitant chemoradiation therapy (CCRT) and was parameterized as RT or CCRT. Human papilloma virus (HPV) tumor status was collected for oropharyngeal and laryngeal tumors, HPV status was evaluated using P16 status as a surrogate [18]. HPV status was categorized as positive, negative and inapplicable. Karnofsky performance status (KPS) score was collected and parameterized as continuous. The number of comorbidities was determined and included the following conditions: respiratory conditions, nervous system disorders, immune system disorders, urinary system disorder, skeletal system disorders, endocrine disorders, if diabetic (diet controlled, insulin controlled, oral medication, other), cardiovascular system disorders, and digestive system disorders.

#### 2.4.3. Body Composition

SMD was considered as a continuous variable for the purposes of linear regression in pretreatment association analysis. For longitudinal analysis, muscle density was dichotomized to aid in interpretability as myosteatosis and normal muscle according to BMI appropriate cut-offs for head and neck cancer as previously described [10,19]. Myosteatosis was defined as <41 Hounsfield units (HU) for those with a BMI in the healthy or underweight range (≤24.9 kg/m^2^) and <33 HU for those with a BMI in the overweight or obese range (≥25.0 kg/m^2^) [10,20].

#### 2.4.4. Social History Characteristics

Partner status was determined through patient self-report and included: married, single, separated, divorced, significant other, widowed, and unknown (or refused to answer). Current smoking status was self-reported and classified as current smoker, former smoker or never smoker. Alcohol consumption was also self-reported; consumption was parameterized as current, former, never or unknown.

### 2.5. Statistical Analysis

Demographic, diagnosis, and treatment data was summarized using descriptive statistics. Continuous variables were summarized as the mean (median) and standard deviation (range) and compared using independent sample *t*-tests, and categorical variables were summarized as frequencies and percentages and compared using chi-square tests.

#### Longitudinal Analyses

The strength of association between pretreatment myosteatosis and each facet of quality of life from pretreatment to one year after treatment completion was analyzed using mixed effects regression modeling establishing the difference in quality of life score between those with myosteatosis compared to those with normal muscle density. Mixed effects modelling with an autoregressive covariance structure was used to account for a linear outcome (QOL) that is repeatedly measured on the same individuals over time. The linear mixed effects model has a number of assumptions which must be met and were tested for, such as: normality of residuals, homogeneity of variance, and lack of multicollinearity in covariates [21]. The following covariates were considered as potential confounders based on the literature: age, race, number of comorbidities, SMI, BMI, TAT, alcohol drinking and smoking status, and partner status. Others were considered: AJCC stage, tumor site, cancer treatment, KPS and HPV status. All potential confounders were tested for inclusion in the final model through statistical analysis. If inclusion in the base model changed the measure of association by 10% or more they were deemed statistical confounders and maintained in the final model; age and length of follow-up was included in all models by default. Analyses were conducted on the whole sample and stratified by sex. All analysis was conducted utilizing SAS (version 9.4, SAS Institute, Cary, NC, USA).

## 3. Results

### 3.1. Population Characteristics

Data from 163 patients were analyzed for this study. Of 171 eligible patients, eight were excluded (4.7%) for lack of pretreatment QOL questionnaires. The study population consisted of 134 men and 29 women (Table 1). The average age of patients was 61.8 ± 9.6 years (66.3 ± 9.6 myosteatosis, 59.5 ± 8.0 normal SMD, *p* < 0.0001). The majority were white (91.4%) and male (82.2%). The average number of comorbidities was 2.3 ± 1.9. Approximately half of the sample were former smokers (54.0%) and current alcohol drinkers (51.5%) with similar distributions between those with myosteatosis and those without.

Those with pretreatment myosteatosis had a higher BMI (29.0 ± 5.7 vs. 28.4 ± 9.0 kg/m^2^, *p* = 0.001) and lower SMI (44.4 ± 8.6 cm^2^/m^2^ vs. 59.9 ± 10.7 cm^2^/m^2^ *p* < 0.0001). The average TAT was 383.2 ± 386.4 cm^2^ (337.1 ± 198.0 cm^2^, myosteatosis and 406.6 ± 142. 0 cm^2^, normal SMD, *p* = 0.02) with the primary contributor of difference being visceral adipose tissue (142.40 ± 94.1 cm^2^, myosteatosis vs. 199.2 ± 89.9 cm^2^, normal SMD, *p* = 0.0002).

The largest group was oropharyngeal cancer (55.8%), followed by laryngeal cancer (23.9%); approximately half had HPV-associated tumors (51.5%). The distribution of AJCC stage from I–IV was as follows: 3.7% at stage I, 36.8% at stage II, 28.2% at stage III, and 31.3% at stage IV. A vast majority of patients were treated with both definitive radiation and chemotherapy (95.1%). The Karnofsky performance status at baseline was six points lower in those with pretreatment myosteatosis compared to those with normal muscle density (*p* = 0.002).

The mean global QOL score was 73.3 ± 22.3 points with no difference between groups (*p* = 0.93). Physical function QOL mean score was 88.3 ± 17.2 with an average score of 82.1 ± 21.2 among those with myosteatosis and 91.5 ± 13.8 in those with normal muscle density (*p* = 0.004).

### 3.2. Longitudinal Associations of Baseline Myosteatosis with QOL Trend

**Figure 1:**
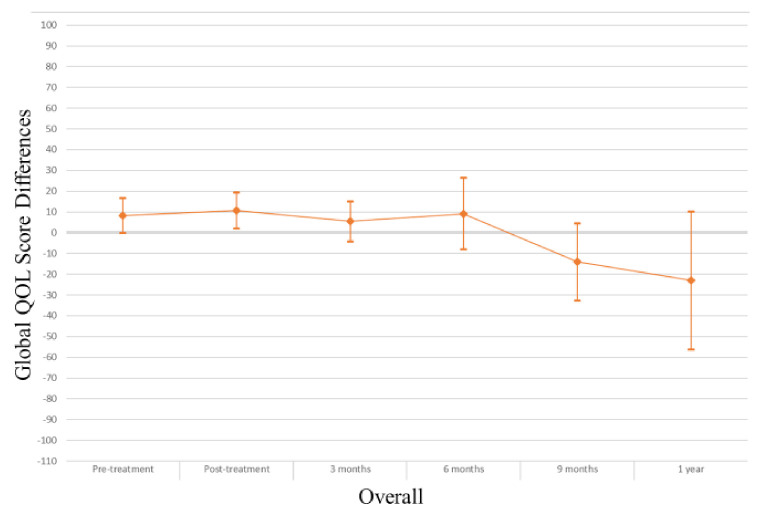
Global QOL score differences in overall study population.

**Figure 2:**
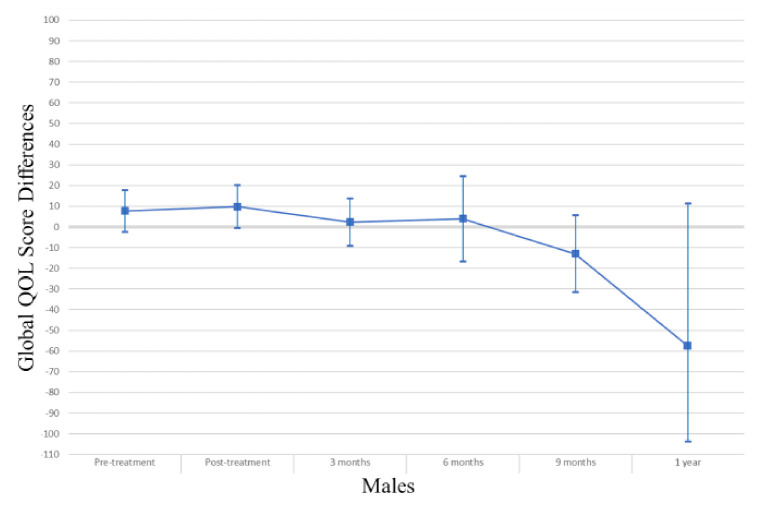
Global QOL score differences in male study population.

**Figure 3:**
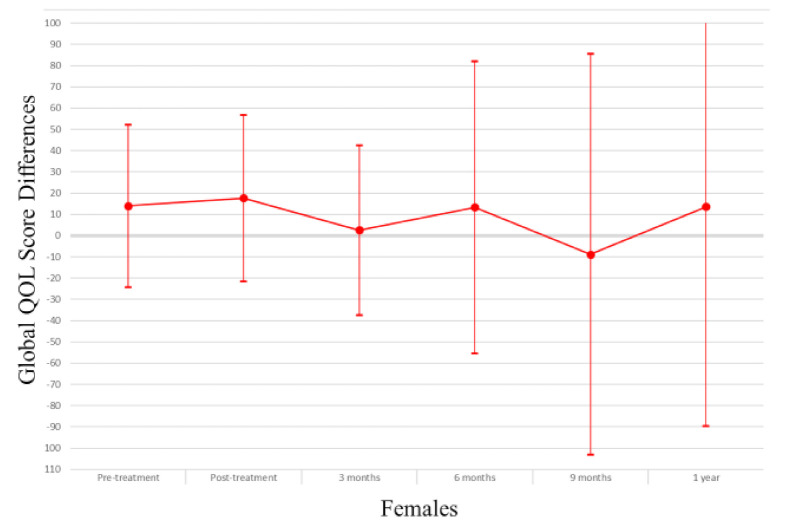
Global QOL score differences in female study population.

In the overall study sample (Table 2), those with myosteatosis had a physical QOL score 9.48 points lower than individuals with normal muscle density at pretreatment (*p* = 0.004), 10.94 points lower at 3 months post treatment (*p* = 0.006) and 31.74 points lower at one year post-treatment (*p* = 0.03). After adjusting for age, muscle mass, KPS, and stage of cancer at diagnosis the association was no longer apparent (pretreatment β −0.74, *p* = 0.84; 3 months β 3.03, *p* = 0.43; one-year β −24.55, *p* = 0.08). Males with myosteatosis had a physical QOL score 9.98 points lower at pretreatment (*p* = 0.01), 12.07 points lower at 3 months post-treatment (*p* = 0.01) and 51.51 points lower at one year following treatment completion (*p* = 0.01) than those with normal muscle density. After adjusting for age, muscle mass, KPS, and stage of cancer at diagnosis, the association was significant at one year post-treatment (β −46.84; *p* = 0.01). Among women, a borderline significant finding occurred in adjusted models at treatment completion where those with myosteatosis scored higher in physical QOL scores (β 19.12; *p* = 0.04). For a visual representation of the adjusted models for global QOL score differences between those with myosteatosis and those with normal musculature, see Figure 1, Figure 2 and Figure 3.

**Figure 4:**
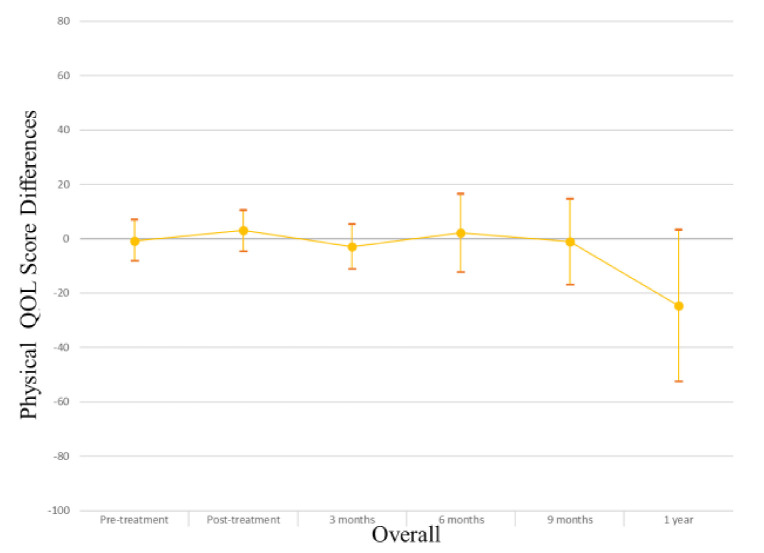
Physical QOL score differences in overall study population.

**Figure 5:**
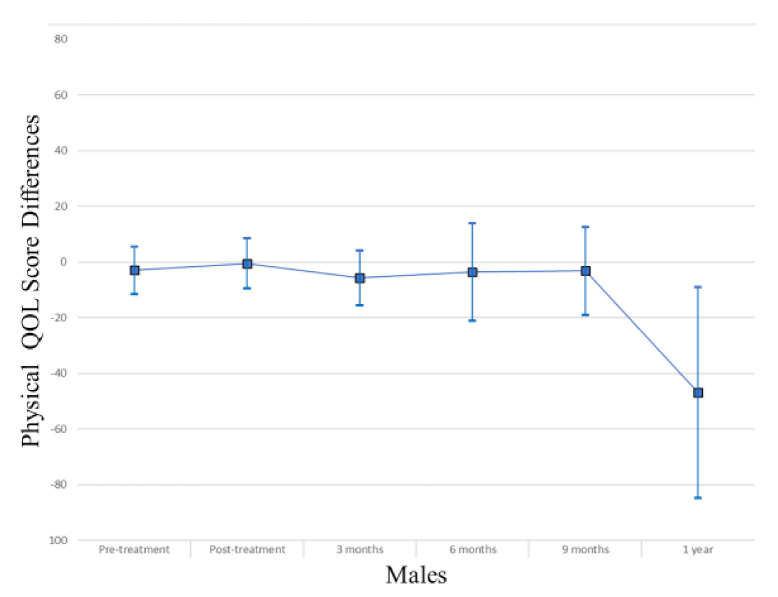
Physical QOL score differences in male study population.

**Figure 6:**
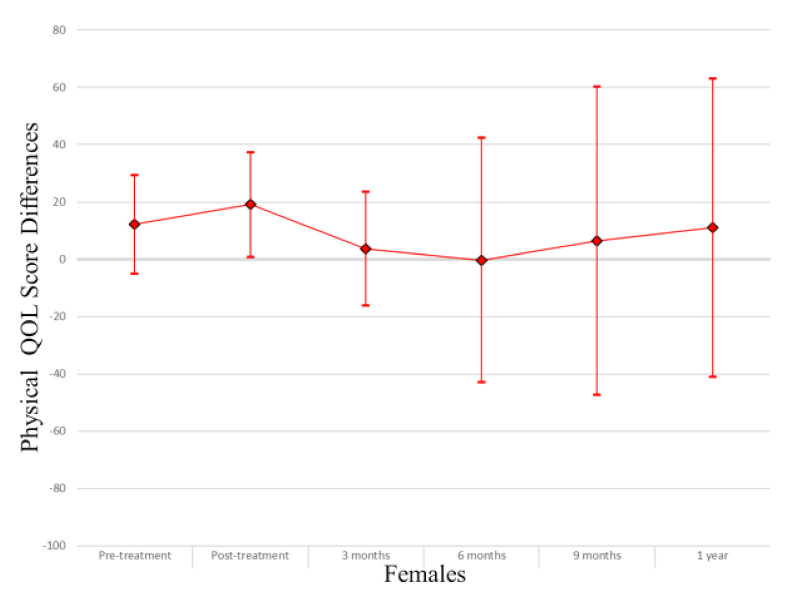
Physical QOL score differences in female study population.

In the overall sample (Table 3), those with myosteatosis had global QOL scores 21.97 points lower than those with normal SMD at 9 months post treatment (*p* = 0.02). After adjustment, just post treatment an improved global QOL score emerged for those with myosteatosis (β 10.67, *p* = 0.02).Among men, global QOL scores were 21.64 points lower in those with myosteatosis at nine months post-treatment (*p* = 0.02) and 54.64 points lower at one year post-treatment (*p* = 0.02). After adjusting for age, body mass index, muscle mass, total adipose tissue, cancer stage, site and treatment, KPS, partner status, smoking status, and alcohol consumption status those with myosteatosis averaged 57.57 points lower at one-year post treatment (*p* = 0.01). For a visual representation of the adjusted models for physical QOL score differences between those with myosteatosis and those with normal musculature, see Figure 4, Figure 5 and Figure 6.

## 4. Discussion

The study demonstrated that men with myosteatosis are more likely to experience decline in QOL scores 12 months after the initial cancer treatment compared to those with normal musculature. The trend was similar in women but the decline in QOL was not statistically significant.

The study sample was similar to HNC populations typical of the United States, where participants are predominately male, non-Hispanic white, with an oropharyngeal primary tumor site [22]. Our measure of myosteatosis was slightly higher than a prior study of myosteatosis and sarcopenia in HNC patients (39.3 vs. 30.5 HU at baseline) although our mean age and sex distribution was similar [10]. Our myosteatosis measure was in accord with other studies of different cancer patients prior to treatment [23,24]. The stage distribution in our sample was expected based on prior literature [25]. The study sample at diagnosis was similar to national averages in BMI which was encouraging given the high rates of sarcopenia prevalent in HNC [26].

It is not surprising that in a small sample myosteatosis was significantly associated with better physical QOL outcomes. Results from a cross-sectional study in Ireland suggest that myosteatosis and sarcopenia are associated with poorer QOL and functional status in multiple cancer types [27]. However, Nakayama et al., in a recent small ovarian cancer study, found no association with either low SMI nor low SMD and cancer survival [28]. The ovarian cancer study may have been underpowered or have suffered from a lack of consistency in measuring cut-offs in Japanese ovarian cancer patients which is mentioned by the authors. A recent study indicated a link between health-related QOL and survival via principal component analysis in HNC [4]. In that study those with a higher QOL score would have a higher principle component score and improved overall survival. Findings from a recent meta-analysis suggest that myosteatosis is prognostic of overall mortality in multiple cancer types [29]. Given the association between baseline myosteatosis and QOL trajectories it may be worth pursuing the direct association between myosteatosis and survival. A recent meta-analysis also indicated the association between low SMI and both poor overall and relapse-free survival in HNC [30]. Findings from our group also show an association between QOL trajectories and changes in a patient’s short performance physical battery score [31]. Each of these tools may serve a useful purpose in helping to determine patients in need of enhanced services over the course of their oncology treatment and survivorship.

Extra assistance could involve exercise or physical therapy interventions. A recent systematic review and meta-analysis among adults indicated that exercise was able to decrease fat infiltration regardless of age at baseline [32]. The Lifestyle Interventions and Independence for Elders (LIFE) pilot study showed that even minor physical activity was associated with lack of age-associated loss of strength even in the midst of loss of muscle mass due to a 17% difference in intramuscular adipose tissue [33]. Exercise has also been shown to decrease loss of HRQOL in cancer populations [34]. Enhanced services could also include nutritional support. A report by Kono et al. among HNC patients showed that early nutritional intervention by a nutritional support team had significantly positive results [35]. Those in the intervention arm experienced less weight loss than those in the nonintervention arm 3.3% vs. 7.3% and had far fewer instances of Grade 3 mucositis (25.0% vs. 70.0%). Further, the preservation of muscle mass and quality through targeted nutritional intervention is key in fighting cancer cachexia [36]. Finally, enhanced services may include supplements or medications. Preclinical studies have indicated the inclusion of fish oil or anti-myostatin antibodies may improve myosteatosis as well as chemotherapy tolerance [37,38]. Therapies still need to be designed and tested but will likely be multimodal involving collaboration between oncology, physical therapy, nutrition, and pharmacy. To this end, there is a current clinical trial underway that is examining exercise therapy in conjunction with nutrition services and the addition of anti-inflammatories in lung and pancreatic cancer patients compared to usual care having been shown previously to be safe and feasible [39].

The study had a number of strengths. Since the population is similar to the make-up of the standard U.S. HNC population, the study results are generalizable to other HNC populations within the U.S being treated by a comprehensive cancer center. The cohort design is a strength which allows for temporality to be established between exposure and outcome, which can help bolster a causal conclusion. Only full-body CT scans were utilized thus improving rigor and reproducibility. Likewise, although only one trained researcher was used to interpret and report on the CT scans which eliminated laboratory drift, findings were audited by three independent investigators. All patients were managed by one radiation oncologist which allowed for consistency in care decisions. 

The study also had some limitations. The study represents patients from one cancer center and therefore is not generalizable to other regions with different care patterns. The study has lower statistical power for women. The study contains patients with multiple HNC locations, however this was controlled for during the analysis when appropriate [40]. The study did not include patients who had undergone surgery. Given the high rate if surgical complications faced by those with sarcopenia it is possible that the poor QOL in this study is underreported [41]. As an observational study there was the possibility of residual confounding due to a lack of randomization. There was both a mix of stages including a lack of statistical control for local vs metastatic disease which could have led to lower QOL scores. There was also a mix of those receiving induction treatment with those who did not. Comorbidities were measured only as a count measure instead of through a validated tool such as Charlson comorbidity index, however the number of comorbidities was equivalent between the study groups and hence did not present as a statistical confounder. There was not fine measurement of smoking or drinking habits which have been previously shown to impact both musculature and QOL. There are unmeasured confounders such as physical activity which may have impacted both musculature as well as both physical and global QOL scores.

## 5. Conclusions

The aim of this study was to explore the association between myosteatosis and QOL from pretreatment through one year following treatment completion. The study showed a statistically significant association between pretreatment muscle density and physical QOL and global QOL in male HNC patients 12 months after treatment completion. These results suggest the need for further exploration of myosteatosis as a therapeutic patient-centered marker in head and neck (and other) cancers throughout the cancer continuum. If these results are replicated, myosteatosis may be a way of determining patients in need of extra assistance over the course of treatment and beyond.

## Figures and Tables

**Table 1:** Patient characteristics stratified by myosteatosis prior to treatment.

Characteristics	All *n* = 163	Myosteatosis *n* = 55 (33.7)	Normal SMD *n* = 108 (62.6)	*p*
Age (years)	61.8 (9.6)	66.3 (9.6)	59.5 (8.0)	<0.0001
Sex				<0.0001
Male	134 (82.2)	33 (60.0)	101 (93.5)	
Female	29 (17.8)	22 (40.0)	7 (6.5)	
Race				0.45
White	149 (91.4)	49 (89.1)	100 (92.6)	
Black	14 (8.6)	6 (10.9)	8 (7.4)	
Comorbidities	2.3 (1.9)	2.5 (1.8)	2.3 (2.0)	0.43
BMI (kg/m^2^)	28.9 (6.4)	29.0 (5.7)	28.4 (9.0)	0.001
SMA (cm^2^)	163.2 (40.5)	128.8 (31.9)	180.7 (32.4)	<0.0001
SMI (cm^2^/m^2^)	54.6 (12.4)	44.4 (8.6)	59.9 (10.7)	<0.0001
VAT (cm^2^)	180.0 (94.9)	142.4 (94.1)	199.2 (89.9)	0.0002
SAT (cm^2^)	190.3 (97.7)	177.3 (124.7)	197.0 (80.5)	0.29
IMAT (cm^2^)	12.8 (8.3)	17.4 (11.6)	10.4 (4.6)	<0.0001
TAT (cm^2^)	383.2 (386.4)	337.1 (198.0)	406.6 (142.0)	0.02
SMD (HU)	39.3 (9.1)	29.6 (6.1)	44.2 (5.9)	<0.0001
Myosteatosis				--
Yes	55 (33.7)	55 (100.0)	0	
No	108 (66.3)	0	108 (100.0)	
Tumor site				0.40
Oral cavity	5 (3.1)	2 (3.6)	3 (2.8)	
Nasopharynx	8 (4.9)	2 (3.6)	6 (5.6)	
Oropharynx	91 (55.8)	25 (45.5)	66 (61.1)	
Hypopharynx	8 (4.9)	4 (7.3)	4 (3.7)	
Larynx	39 (23.9)	17 (30.9)	22 (20.4)	
Salivary	1 (0.6)	1 (1.8)	--	
Other	11 (6.7)	4 (7.3)	7 (6.5)	
AJCC stage				0.19
I	6 (3.7)	2 (3.6)	4 (3.7)	
II	60 (36.8)	14 (25.5)	46 (42.6)	
III	46 (28.2)	18 (32.7)	28 (25.9)	
IV	51 (31.3)	21 (38.2)	30 (27.8)	
KPS	88.90 (10.4)	84.9 (12.8)	90.9 (8.4)	0.002
HPV				0.008
Positive	84 (51.5)	19 (34.6)	20 (18.5)	
Negative	37 (22.7)	17 (30.9)	20 (18.5)	
Inapplicable	42 (25.8)	19 (34.6)	23 (21.3)	
Treatment				0.01
RT only	8 (4.9)	6 (10.9)	2 (1.9)	
RT + chemotherapy	155 (95.1)	49 (89.1)	106 (98.2)	
Smoking status				0.09
Current	28 (17.2)	14 (25.5)	14 (13.0)	
Former	88 (54.0)	29 (52.7)	59 (54.6)	
Never	47 (28.8)	12 (21.8)	35 (32.4)	
Alcohol drinking status				0.09
Current	84 (51.5)	25 (45.5)	59 (54.6)	
Former	40 (24.5)	19 (34.6)	21 (19.4)	
Never	25 (15.3)	5 (9.1)	20 (18.5)	
Unknown	14 (8.6)	6 (10.9)	8 (7.4)	
Partner status				0.04
Married	87 (53.4)	23 (41.8)	64 (59.3)	
Single	44 (27.0)	18 (32.7)	26 (24.1)	
Divorced	18 (11.0)	7 (12.7)	11 (10.2)	
Widowed	6 (3.7)	5 (9.1)	1 (1.0)	
Unknown	8 (4.9)	2 (3.6)	6 (5.6)	
Quality of life				
Global function	73.3 (22.3)	73.0 (23.8)	73.4 (21.8)	0.93
Physical function	88.3 (17.2)	82.1 (21.2)	91.5 (13.8)	0.004

Abbreviations: AJCC, American Joint Committee on Cancer; BMI, body mass index; HU, Hounsfield units; HPV, human papilloma virus; IMAT, intermuscular adipose tissue; KPS, Karnofsky Performance Status; kg, kilograms; s, seconds; RT, radiotherapy; SAT, subcutaneous adipose tissue; SMA, skeletal muscle area; SMD, skeletal muscle density, SMI, skeletal muscle index; TAT, total adipose tissue; VAT, visceral adipose tissue. Data are presented as frequency (percent) and mean (SD).

**Table 2:** Average difference in physical QOL score comparing those with myosteatosis to those with normal skeletal muscle density from pretreatment to one year after treatment.

Overall Population								
**Assessment time**	**Physical**	Beta Coefficient *	**95% CI**	***p***	**Physical**	Beta Coefficient *	**95% CI**	***p***
Pretreatment	Crude model	−9.48	−15.96, −3.00	0.004	Adjusted	−0.74	−7.97, 6.94	0.84
Post-treatment		−5.52	−12.40, 1.35	0.12		3.03	−4.47, 10.52	0.43
3 months post-treatment		−10.94	−20.56, 7.89	0.006		−2.79	−10.98, 5.39	0.50
6 months post-treatment		−6.33	−14.68, 19.33	0.38		2.26	−12.11, 16.62	0.76
9 months post-treatment		−8.24	−24.17, 7.69	0.31		−1.07	−16.74, 14.61	0.89
1 year post-treatment		−31.74	−60.87, −2.61	0.03		−24.55	−52.46, 3.36	0.08
Males								
**Assessment time**	**Physical**	Beta Coefficient *	**95% CI**	***p***	**Physical**	Beta Coefficient *	**95% CI**	***p***
Pretreatment	Crude model	−9.98	−17.68, −2.29	0.01	Adjusted	−2.99	−11.57, 5.58	0.49
Post-treatment		−7.52	−15.70, 0.66	0.07		−0.47	−9.39, 8.45	0.92
3 months post-treatment		−12.07	−21.41, −2.74	0.01		−5.80	−15.62, 4.03	0.25
6 months post-treatment		−9.57	−27.10, 8.00	0.28		−3.65	−21.22, 13.93	0.68
9 months post-treatment		−8.88	−24.82, 7.05	0.27		−3.25	−19.09, 12.59	0.69
1 year post-treatment		−51.51	−90.71, −12.31	0.01		−46.84	−84.79, −8.90	0.01
Females								
**Assessment time**	**Physical**	Beta Coefficient *	**95% CI**	***p***	**Physical**	Beta Coefficient *	**95% CI**	***p***
Pretreatment	Crude model	5.02	−13.52, 23.56	0.59	Adjusted	12.19	−4.99, 29.38	0.16
Post-treatment		12.39	−7.29, 32.07	0.87		19.12	0.89, 37.35	0.04
3 months post-treatment		1.58	−19.84, 23.01	0.88		3.67	−16.21, 23.55	0.71
6 months post-treatment		−6.98	−52.14, 38.18	0.76		−0.28	−42.91, 42.34	0.99
9 months post-treatment		11.35	−48.91, 71.61	0.71		6.51	−47.24, 60.27	0.81
1 year post-treatment		−13.74	−74.33, 46.85	0.65		11.07	−41.03, 63.16	0.67

* Indicates the difference in QOL score between those with myosteatosis and those with normal musculature. For example, a beta coefficient of 3.00 would mean someone with myosteatosis would score on average 3.00 points higher whereas a beta coefficient of −7.35 indicates a score of 7.35 points lower for the person with myosteatosis compared to the person with normal musculature. Crude model contains myosteatosis (exposure) and time. Adjusted model contains adjustment for age, muscle mass, Karnofsky performance status score, and cancer stage.

**Table 3:** Average difference in Global QOL score comparing those with myosteatosis to those with normal skeletal muscle density from pretreatment to one year after treatment.

Overall Population								
**Assessment time**	**Global**	Beta Coefficient *	**95% CI**	***p***	**Global**	Beta Coefficient *	**95% CI**	***p***
Pretreatment	Crude model	−0.35	−7.56, 6.86	0.92	Adjusted	8.27	−0.16, 16.71	0.05
Post-treatment		2.09	−5.63, 9.82	0.59		10.67	1.90, 19.44	0.02
3 months post-treatment		−2.16	−10.96, 6.64	0.63		5.40	−4.19, 14.98	0.27
6 months post-treatment		0.37	−16.35, 17.09	0.97		9.11	−8.06, 26.28	0.30
9 months post-treatment		−21.97	−40.29, −3.65	0.02		−14.06	−32.59, 4.47	0.14
1 year post-treatment		−30.70	−63.56, 2.16	0.07		−23.01	−56.21, 10.19	0.17
Males								
**Assessment time**	**Global**	Beta Coefficient *	**95% CI**	***p***	**Global**	Beta Coefficient *	**95% CI**	***p***
Pretreatment	Crude model	−0.27	−8.97, 8.43	0.95	Adjusted	7.69	−2.29, 17.68	0.13
Post-treatment		1.76	−7.53, 11.04	0.71		9.78	−0.61, 20.16	0.06
3 months post-treatment		−4.40	−15.06, 6.27	0.42		2.39	−9.07, 13.84	0.68
6 months post-treatment		−4.58	−25.01, 15.84	0.66		3.93	−16.71, 24.58	0.71
9 months post-treatment		−21.64	−39.93, −3.35	0.02		−13.02	−31.60, 5.55	0.05
1 year post-treatment		−54.64	−98.88, −9.55	0.02		−57.57	−103.71, 11.42	0.01
Females								
**Assessment time**	**Global**	Beta Coefficient *	**95% CI**	***p***	**Global**	Beta Coefficient *	**95% CI**	***p***
Pretreatment	Crude model	7.68	−12.30, 27.67	0.45	Adjusted	13.96	−24.28, 52.21	0.45
Post-treatment		10.15	−11.73, 32.02	0.36		17.68	−21.44, 56.79	0.35
3 months post-treatment		−4.63	−28.45, 19.19	0.70		2.54	−37.42, 42.51	0.89
6 months post-treatment		4.61	−48.85, 58.08	0.86		13.29	−55.35, 81.92	0.70
9 months post-treatment		16.04	−49.23, 81.30	0.63		−8.84	−103.20, 85.51	0.85
1 year post-treatment		−16.51	−81.66, 48.64	0.61		13.60	−89.73, 116.93	0.79

* Indicates the difference in QOL score between those with myosteatosis and those with normal musculature. For example, a beta coefficient of 3.00 would mean someone with myosteatosis would score on average 3.00 points higher whereas a beta coefficient of −7.35 indicates a score of 7.35 points lower for the person with myosteatosis compared to the person with normal musculature. Crude model contains myosteatosis (exposure) and time. Adjusted model contains adjustment for age, body mass index, muscle mass, total adipose tissue, cancer stage, site and treatment, Karnofsky performance status score, partner status, smoking status, and alcohol consumption status.

## Data Availability

Shaver, Platek and Singh had full access to all the data in the study and take responsibility for the integrity of the data and the accuracy of the data analysis. The data underlying this article cannot be shared publicly for the privacy of individuals that participated in the study. The data are available from the corresponding author upon reasonable request.
